# High seroconversion rate to Rift Valley fever virus in cattle and goats in far northern KwaZulu-Natal, South Africa, in the absence of reported outbreaks

**DOI:** 10.1371/journal.pntd.0007296

**Published:** 2019-05-03

**Authors:** Carien van den Bergh, Estelle H. Venter, Robert Swanepoel, Peter N. Thompson

**Affiliations:** 1 Department of Veterinary Tropical Diseases, Faculty of Veterinary Science, University of Pretoria, Onderstepoort, Gauteng, South Africa; 2 College of Public Health, Medical and Veterinary Sciences, James Cook University, Townsville, Queensland, Australia; 3 Epidemiology Section, Department of Production Animal Studies, Faculty of Veterinary Science, University of Pretoria, Onderstepoort, Gauteng, South Africa; School of Veterinary Medicine University of California Davis, UNITED STATES

## Abstract

**Background:**

Rift Valley fever (RVF) is a mosquito-borne zoonotic disease characterized in South Africa by large epidemics amongst ruminant livestock at very long, irregular intervals, mainly in the central interior. However, the presence and patterns of occurrence of the virus in the eastern parts of the country are poorly known. This study aimed to detect the presence of RVF virus (RVFV) in cattle and goats in far northern KwaZulu-Natal province and to estimate the prevalence of antibodies to the virus and the incidence rate of seroconversion.

**Methodology:**

Cross-sectional studies were performed in communally farmed cattle (n = 423) and goats (n = 104), followed by longitudinal follow-up of seronegative livestock (n = 253) 14 times over 24 months, representing 160.3 animal-years at risk. Exposure to RVFV was assessed using an IgG sandwich ELISA and a serum neutralization test (SNT) and seroconversion was assessed using SNT. Incidence density was estimated and compared using multivariable Poisson models and hazard of seroconversion was estimated over time.

**Principal findings:**

Initial overall seroprevalence was 34.0% (95%CI: 29.5–38.8%) in cattle and 31.7% (95%CI: 22.9–41.6%) in goats, varying by locality from 18–54%. Seroconversions to RVFV based on SNT were detected throughout the year, with the incidence rate peaking during the high rainfall months of January to March, and differed considerably between years. Overall seroconversion rate in cattle was 0.59 per animal-year (95% CI: 0.46–0.75) and in goats it was 0.41 per animal-year (95% CI: 0.25–0.64), varying significantly over short distances.

**Conclusions/Significance:**

The high seroprevalence in all age groups and evidence of year-round viral circulation provide evidence for a hyperendemic situation in the study area. This is the first study to directly estimate infection rate of RVFV in livestock in an endemic area in the absence of reported outbreaks and provides the basis for further investigation of factors affecting viral circulation and mechanisms for virus survival during interepidemic periods.

## Author summary

Rift Valley fever (RVF) is a mosquito-transmitted viral disease that may cause large epidemics in domestic livestock and in humans. Although currently largely confined to Africa, it is of international concern due to its ability to spread and become established in areas where suitable mosquito vectors occur. Outbreaks occur sporadically, associated with conditions favourable for proliferation of mosquito populations, such as high rainfall and flooding, yet their location and timing remain difficult to predict. In other areas there is evidence that RVF virus is endemic and may circulate without causing outbreaks. However, the location and extent of such areas is poorly known, as is the transmission dynamics of the virus in those areas. In this paper, we report the existence of such an area of endemic RVF virus transmission on the tropical coastal plain of South Africa bordering Mozambique, where we found a high rate of exposure of domestic cattle and goats to the naturally circulating virus over a two-year period, with no outbreaks being reported. Research in such areas will help us to assess the potential for spread of the virus to other areas and also to better understand the behaviour of the virus during periods between epidemics.

## Introduction

Rift Valley fever (RVF) is a zoonotic arboviral disease caused by RVF virus (RVFV), a *Phlebovirus* in the family *Phenuiviridae* [1], mainly affecting livestock and humans and transmitted by *Aedes* and *Culex* spp. mosquitoes. Non-vector transmission of RVFV is not considered important in livestock but humans are easily infected by contact with bodily fluids of infected animals or by inhaling infectious particles [2]. Human infection may present in a self-limiting febrile disease with signs including fever, severe headache, malaise, muscle pain and nausea, but in severe cases can result in encephalopathy, haemorrhagic signs, retinopathy and even death. The disease in ruminants may be characterized by necrotic hepatitis but may also be inapparent or mild, and in pregnant animals usually results in abortions and neonatal mortalities [2].

The virus was first isolated in Kenya in 1930 [3], and has been endemic in sub-Saharan Africa ever since. The virus spread beyond sub-Saharan Africa when outbreaks occurred in the Nile delta in Egypt during 1977–1978 causing mortalities in both humans and livestock [4, 5]. It was first recorded in Mauritania and Senegal in West Africa in 1987 [6] and spread beyond mainland Africa, to Saudi Arabia and Yemen in 2000 [7–9], the Comoros in 2007 [10] and Madagascar in 1990 [11]. Three major RVF epidemics have occurred in South Africa, during 1950–1951, 1974–1975 and more recently 2008–2011 [12]. During the 2010 outbreaks a total of 302 human infections were diagnosed with a case fatality rate of 8% [13] and a loss of 19,000 head of livestock was reported. Species such as buffaloes, camels and other wildlife were also affected [14]. These large epidemics have occurred mainly following unusually heavy rains on the relatively dry central plateau of South Africa. However, in the periods between these large epidemics, several smaller outbreaks or isolated cases have occurred both in the interior and in the eastern parts of the country [12].

The fate of the virus during the long interepidemic periods in South Africa is poorly understood. The virus has been isolated from newly emerged, unfed *Aedes lineatopennis* (*Ae*. *mcintoshi*) in East Africa, suggesting that it may survive extended periods in aedine mosquito eggs, which are very resilient in dry conditions [15]. Romoser et al. [16] provided laboratory evidence in support of transovarial transmission in artificially infected *Ae*. *mcintoshi*, and it remains the most popular theory for interepidemic survival of RVFV; however, evidence for its occurrence in nature is limited. Other possibilities are the occurrence of low-level interepidemic circulation of virus between vectors and unknown hosts in endemic areas, or disappearance of virus from an area with later re-introduction from an endemic area via movement of infected hosts or vectors [2].

One such endemic area may be the low-lying, tropical eastern part of southern Africa, including Mozambique and small parts of north-eastern South Africa. Very few outbreaks have been reported in Mozambique, yet there is serological evidence of widespread exposure to RVFV in livestock [17] and humans [18]. RVFV was responsible for a small outbreak in goats in the Maputo Province, close to the South African border, in 2014 [19]. Recently, the overall seroprevalence of RVFV antibodies in seven provinces of Mozambique was reported to be 37% in cattle, 30% in African buffalo (*Syncerus caffer*), and 29% in domestic ruminants in the Maputo Province [20]. In South Africa, RVFV was isolated from *Ae*. *circumluteolus* in the tropical coastal region of far northern KwaZulu-Natal (KZN) in 1955 [21] and at the same time neutralizing antibodies to RVFV were found in 12% of domestic livestock [22] and 10% of humans [23]. However, subsequent efforts to detect the virus in mosquitoes in that region were unsuccessful [24]. There have also not been any reports of clinical cases of RVF in that region, although further south along the KZN coast there was some evidence of RVFV circulation and sporadic clinical cases in 1972–1973 [25] and in a dairy herd in 1981 [26].

Various reports have inferred the occurrence of interepidemic circulation of RVFV on the basis of seropositivity in the absence of reported outbreaks or vaccination, in livestock [17, 27, 28] wildlife [29, 30] and humans [31, 32]. Two recent studies have reported longitudinal follow-up of sentinel sheep and goats in Kenya, reporting low rates of seroconversion in areas that had previously experienced outbreaks [33, 34]. However, no published studies have attempted to quantify the rate of seroconversion in livestock by follow-up of seronegative animals in a region where outbreaks have never been reported.

The objectives of this study were to determine whether there was evidence of recent circulation of RVFV in the tropical region of far northern KwaZulu-Natal, South Africa, to estimate the seroprevalence in cattle and goats, and to estimate the incidence rate and patterns of seroconversion in domestic ruminants over a two-year period.

## Materials and methods

### Ethics statement

The study protocol was approved by the Animal Ethics Committee of the University of Pretoria (V013-16) and the Department of Agriculture, Forestry and Fisheries, Republic of South Africa, and adhered to the specifications of the South African National Standard (SANS 10386–2008): “The Care and Use of Animals for Scientific Purposes”. Support for the project was obtained from the Director of Veterinary Services of KwaZulu-Natal and the State Veterinarian: Jozini. Verbal consent was obtained from animal owners after the aims of the study were explained to them by the State-employed animal health technicians. Farmers were rewarded for their participation by providing them with small amounts of animal health products such as wound aerosol, topical treatments and endoparasiticides.

### Study area

**Fig 1:**
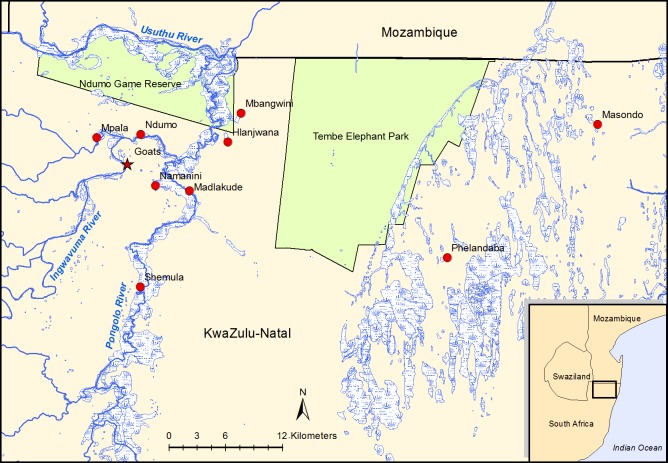
A map of the study area showing major rivers and temporary wetlands, with the locations of the nine diptanks where cattle were sampled and the area where goats were sampled for the cross-sectional study. The map was constructed for this manuscript in Esri ArcGIS 10.2 using country boundaries from Esri ArcGIS Online, diptank coordinates collected during the study, and river [39], wetland [40] and protected area boundary [41] data available under a Creative Commons Attribution (CC BY 4.0) license.

**Fig 2:**
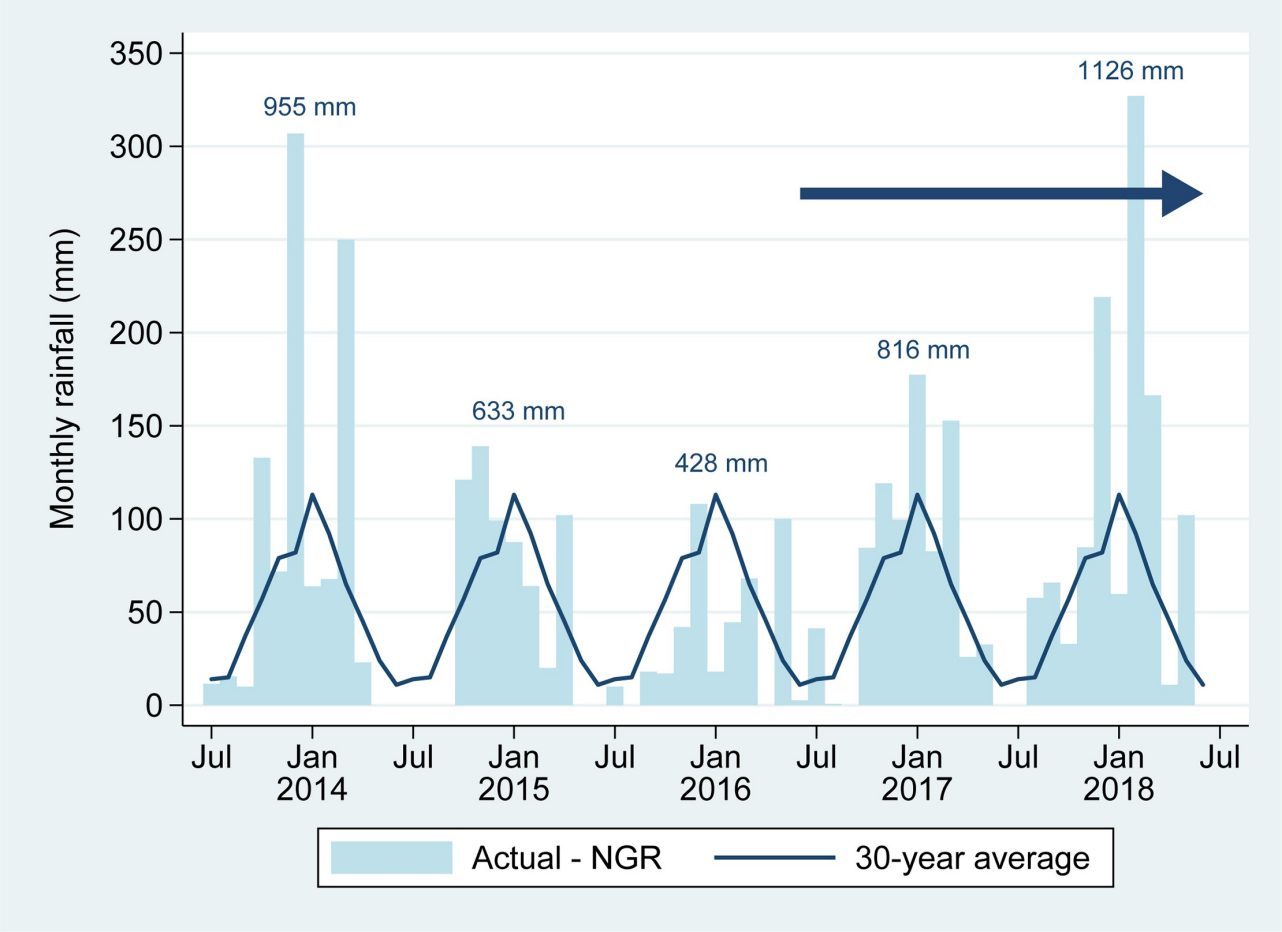
Actual monthly rainfall recorded at Ndumo Game Reserve (NGR) in the study area, with 30-year average. Totals above the bars indicate rainfall for each year (July—June). The blue arrow indicates the study period (June 2016—June 2018).

The study was conducted on the Maputaland coastal plain in far northern KwaZulu-Natal just south of the Mozambique border, an area with a tropical climate [35] characterised by warm, dry winters and hot, wet summers (Fig 1). In the Pongolo River floodplain, the core of the study area, the summer temperatures range from 23–40°C, winter temperatures range from 16–26°C and mean annual rainfall is 600–800 mm [36]. The northwards-flowing Pongolo River forms a floodplain at an elevation of 30–50 m above sea level, approximately 10–15 km to the east of the foothills of the Lebombo Mountains, which rise to about 650 m above sea level. The study area contains a mosaic of bushland, thicket, wooded grassland, riverine forest and floodplain vegetation [37]. Depending on rainfall, seasonal flooding of the Pongolo, Ingwavuma and Usuthu Rivers inundates about 13,000 ha of floodplain, filling numerous pans, some of which retain water during the dry season [38]. The construction of the Pongolapoort Dam in 1973 largely disrupted this cycle, with flooding only occurring with heavy rainfalls or with periodic opening of sluice gates. In very dry years, permanent water may only be present in the Pongolo and Usuthu Rivers and in very few of the larger pans. This study started during a drought period when most of the pans were dry (Fig 2).

Livestock in the study area (mainly cattle and goats) belong to numerous small-scale farmers and are communally grazed. Animals range widely during the day, during the dry season foraging mainly in the floodplains, and may be confined in pens at night. Much of the area lies within a foot-and-mouth disease (FMD) protection zone and cattle are brought weekly (summer) or every two weeks (winter) to diptanks where they are inspected by animal health technicians for signs of FMD and plunge-dipped for ectoparasite control using amitraz. For this inspection purpose, most cattle are individually identified using ear tags. The only vaccinations applied are those provided by the State, namely brucellosis, anthrax, blackquarter and sometimes lumpy skin disease vaccines.

### Study design and sampling

The study took the form of an initial cross-sectional survey of individually identified cattle at nine diptanks (Fig 1) during June 2016, followed by a longitudinal follow-up of seronegative cattle to detect and quantify seroconversion. The nine diptanks were selected on the basis of their location adjacent to the floodplain of the Pongolo River and its tributaries (seven diptanks) and the eastern coastal plain (two diptanks), as well as the willingness of farmers and animal health technicians to participate.

Of the seven diptanks in the western part of the study area, all were situated within 1.5 km of the Pongolo River, except for Mpala which was adjacent to a small tributary which held water for almost the entire study period and Ndumo which was about 4 km from the Pongolo River and adjacent to the Ingwavuma River which remained dry throughout the study period. The two diptanks in the eastern part of the study area were situated in a very sandy area with a mosaic of grassland and bush and with numerous small temporary pans during the wet season.

Sample size (*n*) was calculated in order to detect a seroprevalence (*P*_*exp*_) of 25% with an allowable error (*d*) of 10% and 95% confidence, using the formula *n* = 1.96^2^ *P*_*exp*_(1 –*P*_*exp*_) / *d*^2^ [42]. The required sample size of 73 was then multiplied by a design effect (*D*) of 5.9, calculated by assuming an intracluster correlation coefficient (*ρ*) of 0.1 and a cluster size (*m*) of 50 animals per diptank, using the formula *D* = 1 + *ρ*(*m*– 1) [43]; the overall required sample size was therefore 432.

Animals were selected using systematic random sampling, selecting 2–5 animals per farmer at each diptank as they passed through a crush either before or after dipping. Only cattle >6 months old were sampled. Following serological testing of samples from the cross-sectional study, as many seronegative animals as possible, as well as a small number of seropositive animals, were then re-sampled during 13 sampling episodes at 1-2-month intervals between November 2016 and June 2018. To compensate for loss to follow-up, some additional cattle from the same owners were recruited at various times during the study.

A smaller study of the same type was conducted in goats in an area close to one of the diptanks (Fig 1); 104 goats belonging to seven farmers were sampled during February to April 2017 and seronegative animals were re-sampled at 1–2 monthly intervals until June 2018.

Blood samples were collected from the jugular vein of goats and coccygeal vein of cattle. Samples were refrigerated and transported to a BSL2+ laboratory at the Faculty of Veterinary Science, University of Pretoria, where they were centrifuged. The sera were inactivated at 56°C for 1 hour and stored at -20°C until used.

### Serology

All cattle serum samples that were collected for the cross-sectional study were tested for RVFV IgG antibodies using an IgG sandwich enzyme-linked immunosorbent assay (ELISA) with reported sensitivity of 96.3% and specificity of 99.7% in cattle [44]. In addition, approximately every second sample (due to economic constraints) was tested using the serum neutralization test (SNT). Where possible, samples that tested positive for RVFV IgG were also tested for IgM antibodies using an IgM capture ELISA with reported sensitivity of 100% in sheep and specificity of 99.2% in cattle [44]. In order to detect seroconversion, all samples collected during the longitudinal study, as well as all goat serum samples, were tested using SNT.

Heat-inactivated serum samples were tested by the IgG sandwich ELISA using a previously described and validated method [44]. F96 Maxisorp Immunoplates (AEC-Amersham) were coated with anti-RVF hyperimmune mouse ascitic fluid. Optical densities (OD) were measured at 450 nm. The net OD values were first recorded for each serum as the value determined with the RVFV antigen minus the value determined with the control antigen and subsequently converted into percentage of the OD value of a high positive control serum. A cut-off value optimized for this assay (PP value ≥30) was used.

The IgM capture ELISA was performed following a method previously described [44]. Briefly: plates were coated overnight at 4°C with 100 μl rabbit anti-sheep IgM. OD was determined at 450 nm. The PP was determined as above and an optimized cut-off value of PP≥30 was used.

The SNT was performed in 96-well plate (AEC-Amersham) format according to the standard protocol of the Virology Section, Department of Veterinary Tropical Diseases, Faculty of Veterinary Science, University of Pretoria, which follows the method prescribed by the World Organisation for Animal Health [45]. Briefly: Sera were diluted 1:5 in PBS+ (phosphate buffered saline with added MgCl and CaCl) and two-fold dilutions of the serum were made. The TCID_50_ was determined using the Karber method [46]. A volume of 100 TCID_50_ virus (Smithburn vaccine strain) was added to each dilution and incubated at 37°C for 60 min. A total of 80 μl of African green monkey kidney cells (Vero) (480,000 cells/mL) in MEM containing 5% foetal calf serum (Biowest, Celtic) was added to each well. The microplates were incubated at 37°C in an atmosphere containing 5% CO_2_ and observed daily for cytopathic effect; the titre was taken as the dilution at which 50% of the cells were affected. Results were only accepted if all controls gave the expected results (virus control, positive serum and negative serum). A serum dilution of ≥1:10 was used to define seropositivity.

### Statistical analysis

For samples tested during the cross-sectional study using both the IgG ELISA and the SNT, sensitivity and specificity of the IgG ELISA were estimated, with exact binomial 95% confidence intervals, using the SNT as the gold standard. For animals on which both tests were used, the SNT result was used to define seropositivity, otherwise the IgG ELISA result was used. The RVFV seroprevalence in cattle in June 2016 was calculated overall and by diptank, age group (≤2 y, 2.5–3.5 y, 4–5.5 y, ≥6 y) and sex. For goats the seroprevalence in February 2017 was calculated overall and by age group and sex. Exact binomial 95% confidence intervals were calculated and seroprevalence was compared between diptanks (cattle only), age groups and sexes using multiple logistic regression to control for confounding.

The occurrence of seroconversion was defined as a seronegative result (either during the cross-sectional study as defined above, or during subsequent testing by SNT) followed by a sample from the same animal that tested positive by SNT at a serum dilution of 1:10 or greater. The incidence rate of seroconversion over the 24-month study period (16 months in goats) was then calculated by including all animals that were sampled more than once (irrespective of date of first sampling) and that were seronegative on the first sampling. For the animals that did not seroconvert, animal-time at risk was calculated as the interval between the first and last tests. For animals that did seroconvert, the date of seroconversion was unknown, but was assumed to be the midpoint between the last negative and first positive test. The seroconversion rate was calculated as the number of seroconversions divided by the total animal-years at risk observed, with Poisson exact 95% confidence limits. Estimates were calculated overall for each species, separately for each year of the study (June 2016 to June 2017 and July 2017 to June 2018) and for cattle also separately for each diptank. Associations of age, sex and place with incidence rate were then assessed by including these variables in a multiple Poisson regression model, using animal-time at risk as the exposure variable.

For each species, the seroconversion rate with 95% confidence interval was plotted over time as the derivative of the kernel-smoothed Nelson-Aalen cumulative hazard estimator, using a 60-day kernel width. For this purpose, instead of the midpoint-imputed seroconversion date used above, a randomly selected date between the last negative and first positive test was used for seroconverting animals in order to avoid systematic bias in incidence rate estimation over time [47]. All statistical analyses were done using Stata 15 (StataCorp, College Station, TX, U.S.A.) and statistical significance was assessed at *P*<0.05. Risk surfaces for seroprevalence and seroconversion rate in the study area were created by interpolation using ordinary kriging in ArcGIS 10.2 (Esri, Redlands, CA, U.S.A.).

## Results

A total of 423 cattle was sampled from nine diptanks in June 2016, of which 131 (31.0%) were positive by IgG ELISA. Of 241 cattle tested using SNT, 87 (36.1%) were positive; of these, 75 were IgG ELISA-positive, giving an estimated sensitivity for the IgG ELISA of 86.2% (95% CI: 77.1–92.7%). Of 154 SNT-negative animals, 152 were IgG ELISA-negative, giving an estimated specificity of 98.7% (95% CI: 95.4–99.8%).

### Seroprevalence

The seroprevalence in cattle sampled from nine diptanks in June 2016, based on IgG ELISA and SNT, was 34.0% (144/423; 95% CI 29.5–38.8%). Seroprevalence within diptanks varied from 18% to 54% and it was high in all age groups (Table 1). The multiple logistic regression model (S1 Table) showed significant differences between diptanks, with the odds of seropositivity at Mbangwini (Odds ratio (OR) = 4.5; 95% CI: 1.8–11; *P* = 0.001), Phelandaba (OR = 4.6; 95% CI: 1.8–12; *P* = 0.001) and Shemula (OR = 5.8; 95% CI: 2.3–15; *P*<0.001) being higher than at Madlakude. Odds of seropositivity was the lowest in the 2–4 y age group, being significantly higher in the 4–6 y age group (OR = 2.4; 95% CI: 1.3–4.4; *P* = 0.006), tending to be higher in animals >6 y (*P* = 0.095), and tending to be higher in females than in males (*P* = 0.064). Of the 144 seropositive animals, 114 were tested using the IgM ELISA and 5 (4.4%) were positive; this included 2/6 (33%) of samples that were SNT-positive but IgG ELISA-negative. Therefore the estimated prevalence of IgM-positive animals in the general population was 1.3% (5/393; 95% CI: 0.4–2.9%).

**Table 1:** Rift Valley fever seroprevalence in cattle at diptanks in far northern KwaZulu-Natal, June 2016.

Variable	n	Seroprevalence (%)*	95% CI
**Diptank**			
Madlakude	50	18	7–29
Masondo	21	19	2–36
Mpala	52	25	13–37
Namaneni	50	26	14–38
Ndumo	50	30	17–43
Hlanjwana	50	34	21–47
Phelandaba	50	44	30–58
Mbangwini	50	48	34–62
Shemula	50	54	40–68
**Age (years)**			
<2	56	34	22–46
2 –<4	96	23	15–31
4–6	157	39	31–47
>6	114	34	25–43
**Sex**			
female	331	36	31–41
male	92	27	18–36
**Total**	423	34.0	29.5–38.0

* Based on IgG ELISA, confirmed by serum neutralization test in 241/423 samples.

**Fig 3:**
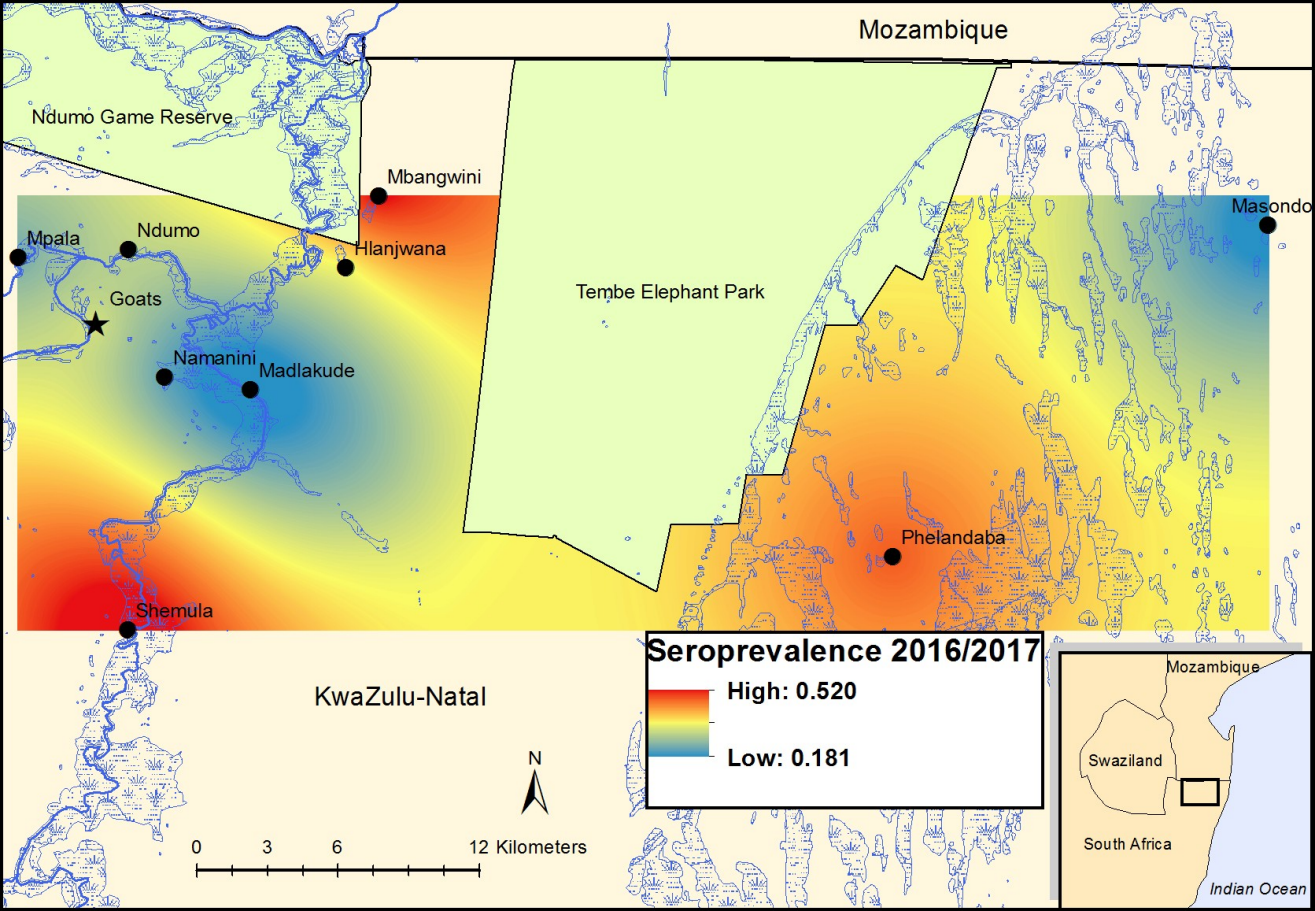
Rift Valley fever virus seroprevalence in cattle and goats in far northern KwaZulu-Natal determined using IgG ELISA and serum neutralization test: risk surfaces created by interpolation using ordinary kriging. The map was constructed for this manuscript in Esri ArcGIS 10.2 using country boundaries from Esri ArcGIS Online, diptank coordinates collected during the study, and river [39], wetland [40] and protected area boundary [41] data available under a Creative Commons Attribution (CC BY 4.0) license.

The seroprevalence in goats sampled during February—April 2017, determined using SNT, was 31.7% (33/104; 95% CI: 22.9–41.6%) (Table 2). Although seroprevalence appeared to increase with age, this was not statistically significant (*P* = 0.549) in the multiple logistic regression model (S2 Table), nor was there a significant difference between sexes (*P* = 0.139). The overall seroprevalence in cattle and goats varied substantially across the study area (Fig 3), with foci of high seroprevalence along the Pongolo River, but was not clearly linked to the presence of wetlands.

**Table 2:** Rift Valley fever seroprevalence in goats in far northern KwaZulu-Natal, February-April 2017.

Variable	n	Seroprevalence (%)*	95% CI
**Age (years)**			
0.5–1.5	25	24	9–45
>1.5–3.5	36	28	14–45
>3.5	43	40	25–56
**Sex**			
female	70	36	25–48
male	28	18	6–37
**Total**	104	31.7	22.9–41.6

* Based on serum neutralization test.

### Seroconversion rate

Of the 279 cattle that were seronegative in June 2016 by IgG ELISA, or by ELISA and SNT if both tests were used, 103 were re-sampled between one and eight times during the study period. An additional 91 SNT-seronegative cattle were recruited at various times during the study period and re-sampled at least once, therefore 194 initially seronegative animals were sampled at least twice over periods ranging between 28 and 721 days. Assuming that seroconversions occurred, on average, midway between the last negative and first positive test, a total of 111.8 animal-years at risk were observed. Due to various factors, including logistical reasons and varying farmer compliance, the majority of animals (167/194) were followed up at four of the diptanks (Namaneni, Mpala, Shemula and Ndumo), with very few followed up at Hlanjwana (13), Madlakude (8) and Masondo (6) and none at Mbangwini and Phelandaba.

**Fig 4:**
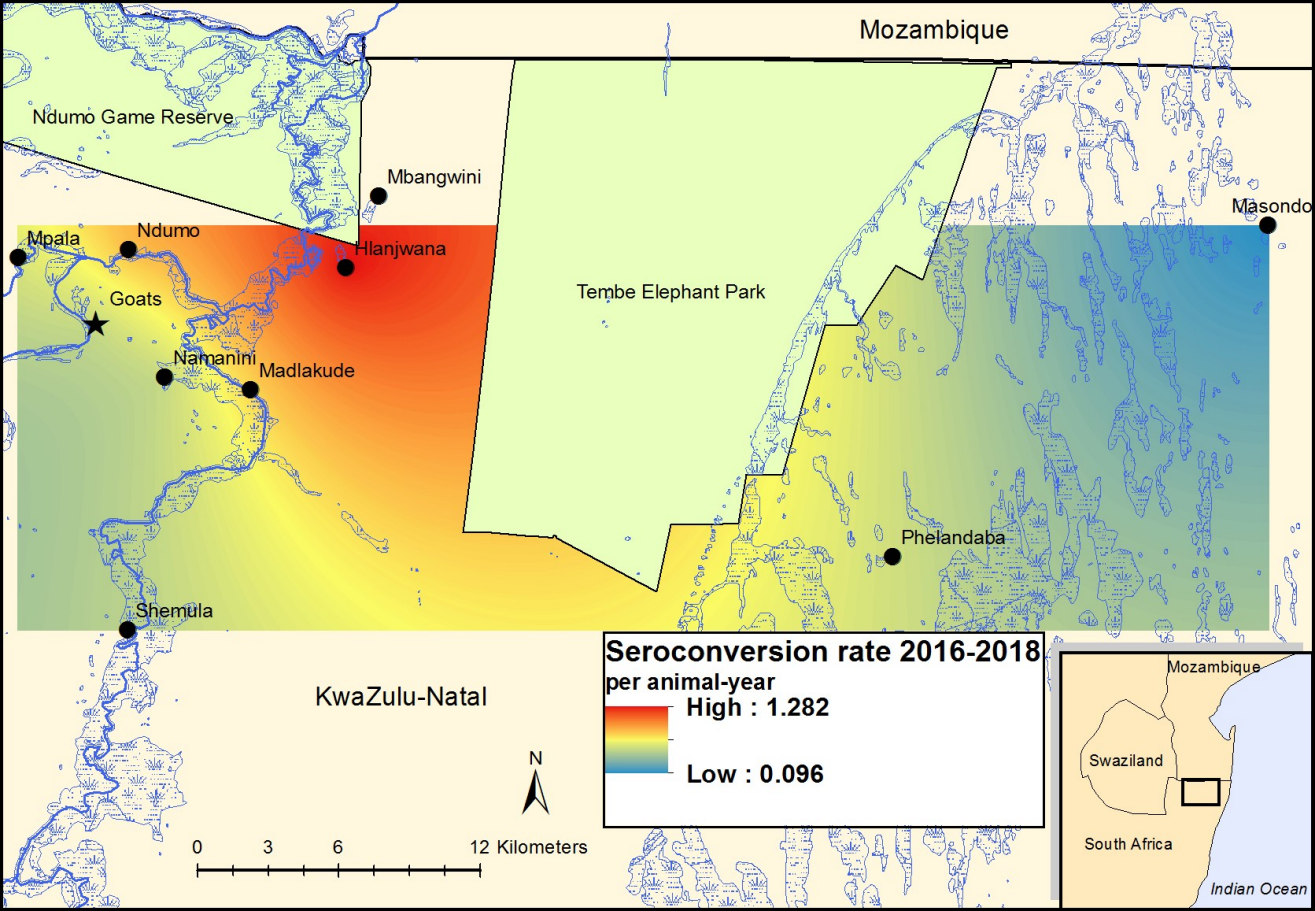
Rift Valley fever virus seroconversion rate in cattle and goats in far northern KwaZulu-Natal between June 2016 and June 2018, determined by serum neutralization test: Risk surfaces created by interpolation using ordinary kriging. The map was constructed for this manuscript in Esri ArcGIS 10.2 using country boundaries from Esri ArcGIS Online, diptank coordinates collected during the study, and river [39], wetland [40] and protected area boundary [41] data available under a Creative Commons Attribution (CC BY 4.0) license.

Seroconversion, determined by SNT, occurred at some stage during the study period in 66 (34%) of initially seronegative cattle. Seroconversions were observed during every interval between samplings for the duration of the study, and at every diptank followed except Masondo, where only six animals were followed for five months each. The overall incidence rate of seroconversion in cattle between June 2016 and June 2018 was 0.59 seroconversions per animal-year (95% CI: 0.46–0.75) (Table 3). Seroconversion rate varied between diptanks, with the lowest rate recorded at Namaneni (0.35 per animal-year) and the highest rates observed at Hlanjwana (1.50 per animal-year) and Ndumo (1.01 per animal-year) (Fig 4). There was no clear relationship at diptank level between the initial seroprevalence in 2016 and the subsequent rate of seroconversion, and the correlation between the two outcomes was not significant (Spearman’s *ρ* = 0.29; *P* = 0.535).

**Table 3:** Incidence rate of seroconversion* to Rift Valley fever virus in cattle and goats in far northern KwaZulu-Natal between June 2016 and June 2018, expressed as numbers of seroconversions per animal-year.

**CATTLE**	**Year 1 (June 2016 –June 2017)**			**Year 2 (July 2017 –June 2018)**			**Total**		
	Animal-years at risk†	Incidence rate	95% CI	Animal-years at risk†	Incidence rate	95% CI	Animal-years at risk†	Incidence rate	95% CI
**Diptank**									
Mpala	16.87	0.83	0.45–1.39	11.09	0.18	0.02–0.65	27.96	0.57	0.33–0.93
Ndumo	11.17	1.34	0.75–2.21	6.64	0.45	0.09–1.32	17.81	1.01	0.60–1.60
Namaneni	16.44	0.36	0.13–0.79	15.00	0.33	0.11–0.78	31.44	0.35	0.17–0.63
Shemula	13.74	0.73	0.35–1.34	10.64	0.19	0.02–0.68	24.37	0.49	0.25–0.86
Hlanjwana	3.25	1.84	0.68–4.01	0.75	0.00	0.00–4.90	4.01	1.50	0.55–3.26
Madlakude	3.74	0.80	0.17–2.35	0	-	-	3.74	0.80	0.17–2.35
Masondo	2.41	0.00	0.00–1.53	0	-	-	2.41	0.00	0.00–1.53
**Total**	**67.62**	**0.80**	**0.60–1.04**	**44.13**	**0.27**	**0.14–0.47**	**111.75**	**0.59**	**0.46–0.75**
**GOATS**	**Year 1 (February–June 2017)**			**Year 2 (July 2017 –June 2018)**			**Total**		
	Animal-years at risk†	Incidence rate	95% CI	Animal-years at risk†	Incidence rate	95% CI	Animal-years at risk†	Incidence rate	95% CI
**Total**	**14.20**	**0.92**	**0.49–1.57**	**34.32**	**0.20**	**0.08–0.42**	**48.52**	**0.41**	**0.25–0.64**

* Seroconversion defined as a previously seronegative animal becoming seropositive based on the serum neutralization test at a serum dilution of 1:10 or greater.

† Seroconversion assumed to have occurred midway between the last negative and the first positive test.

**Fig 5:**
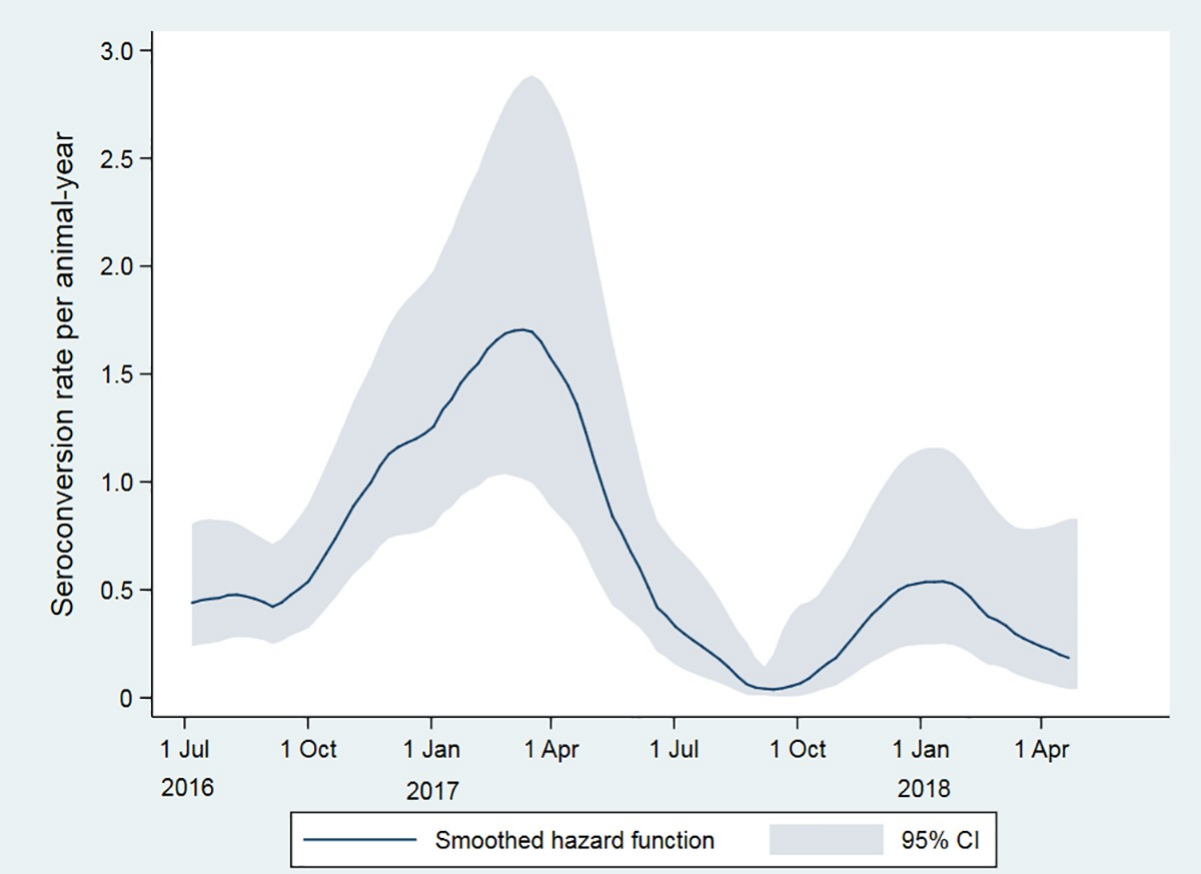
Seroconversion rate of cattle to Rift Valley fever virus in far northern KwaZulu-Natal between June 2016 and June 2018, determined by serum neutralization test and expressed as numbers of seroconversions per animal-year, plotted using the derivative of the kernel-smoothed Nelson-Aalen cumulative hazard estimator.

There was a marked variation in the estimated rate of seroconversion in cattle over time, with the rate being lower during the second year of the study (Table 3). Seroconversion rate increased from October 2016 to peak at just over 1.5 per animal-year between February and April 2017, dropping to almost zero during August-September 2017 and then showing a lower peak of just over 0.5 per animal-year during December-January 2018 (Fig 5).

**Fig 6:**
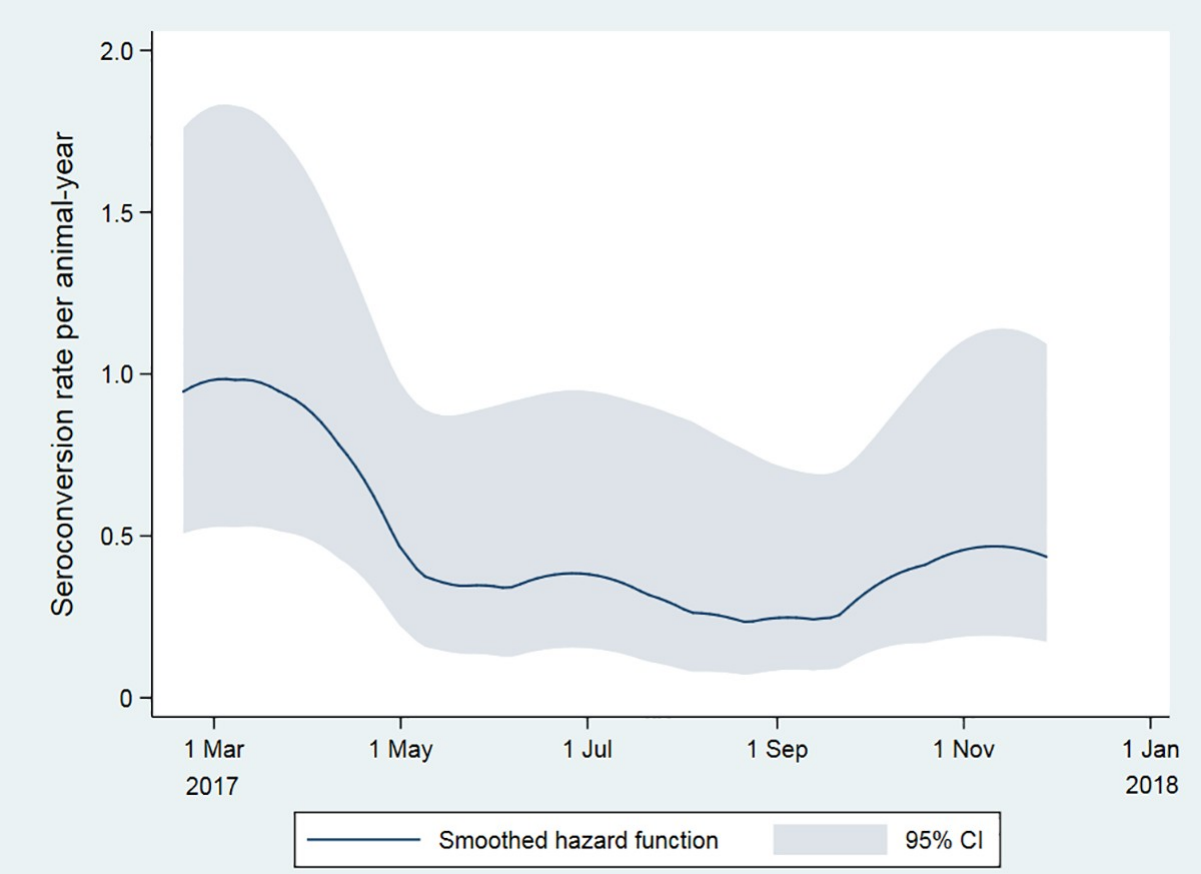
Seroconversion rate of goats to Rift Valley fever virus in far northern KwaZulu-Natal between February and December 2017, determined by serum neutralization test and expressed as numbers of seroconversions per animal-year, plotted using the derivative of the kernel-smoothed Nelson-Aalen cumulative hazard estimator.

Of the goats that were initially seronegative, 95 were followed up for periods of time ranging between 28 and 482 days, of which 20 (21%) seroconverted. A total of 48.5 animal-years at risk were observed. The overall incidence rate of seroconversion in goats between February 2017 and June 2018 was 0.41 seroconversions per animal-year (95% CI: 0.25–0.64) (Table 3) and was also markedly lower during the second summer of the study (Fig 6). The seroconversion rate is not shown after December 2017 since no further seroconversions were observed in the study animals.

For cattle and goats combined, the overall rate of seroconversion was 0.54 per animal-year (95% CI: 0.43–0.66), and was 0.82 per animal-year (95% CI: 0.63–1.04) during the first year and 0.24 per animal-year (95% CI: 0.15–0.38) during the second year. The associations of species, sex, study year and place with seroconversion rate in the multiple Poisson regression model are shown in Table 4. The rate did not differ significantly between species and sexes. However, the rate during the second study year was only a third of that during the first year (*P*<0.001), and significant differences were observed between locations, with Ndumo and Hlanjwana showing higher rates than Namaneni, where the lowest rate was recorded.

**Table 4:** Factors associated with incidence rate of seroconversion to Rift Valley fever virus in cattle and goats in far northern KwaZulu-Natal between June 2016 and June 2018.

Variable and level	Incidence rate ratio	95% CI	*P*-value
**Species**			
Cattle	1*	-	-
Goats	1.62	0.77–3.41	0.206
**Sex**			
Female	1*	-	
Male	0.64	0.36–1.14	0.131
**Year**			
2016–2017	1*	-	-
2017–2018	0.31	0.18–0.53	<0.001
**Place**			
Namanini	1*	-	-
Mpala	1.48	0.69–3.19	0.318
Ndumo	2.39	1.11–5.13	0.025
Hlanjwana	3.01	1.10–8.21	0.031
Madlakude	1.46	0.40–5.26	0.566
Shemula	1.40	0.62–3.19	0.417
Masondo	0.00	0.00 - ∞	0.977

* Reference level

## Discussion

This study showed evidence of a high level of exposure to RVFV in the study area, demonstrating active circulation of RVFV in livestock not associated with reported outbreaks, and to our knowledge is the first published study to quantify directly the seroconversion rate in livestock using the gold-standard SNT. Previous studies to detect interepidemic circulation have inferred low-level viral transmission in epidemic-prone areas by ELISA-based detection of IgG or IgM.

The study was done in an area of South Africa where outbreaks of RVF have never been reported, although occasional small outbreaks and suspected cases had occurred in the more temperate inland and coastal areas to the south of the study area between 1972 and 1986 [12, 25, 26]. Elsewhere in Africa, endemic circulation of RVFV is known or suspected to occur in many areas. Limited numbers of RVFV infections were confirmed to occur in sheep and cattle every year during a seven year interepidemic period in Zimbabwe [48]. A seroprevalence of 33% in cattle and 26% in goats was reported during an interepidemic period in outbreak-prone Garissa County, Kenya during 2013, six years after the last major outbreak [49]. Along the Nile River in Sudan, where outbreaks also occur, a prevalence of 33% in cattle and 34% in goats was found [50]. In Mauritania, also in an area that had experienced outbreaks, a seroprevalence of 15% was detected in cattle and 4% in small ruminants, and IgM and viral RNA was detected in one bovine [51]. A series of surveys in Mayotte found seroprevalences of up to 37% in cattle in the absence of reported outbreaks [52]. A seroprevalence of 17% was reported in Rwanda, also in the absence of reported outbreaks [53]. In Maputo Province, Mozambique, neighbouring our study area to the north, a survey in 2010–2011, during the large outbreak in the central interior of South Africa, found a seroprevalence in cattle of 37%, similar to that in our study [54]. This was followed in 2014 by the detection of a small outbreak of abortion in goats due to RVFV, less than 100 km north of our study area and in the same ecological zone [19]. The 1.3% IgM seroprevalence in this study was comparable to that reported in other endemic areas, e.g. 0.5% in Tanzania [28] and 0–4% in Mayotte [52], indicating the presence of low numbers of recent infections. The seroprevalence in the current study was therefore similar to or higher than those reported elsewhere in Africa where interepidemic circulation is known or suspected to occur and is consistent with ongoing or endemic circulation of RVFV with low numbers of clinical cases likely occurring but overlooked, misdiagnosed or unreported.

Seroprevalence was high in all age groups throughout the study area. Numerous studies have shown that seroprevalence is usually higher in older animals [17, 28, 55, 56], although in northern Somalia it was higher in younger animals [57]. The high seroprevalence in young animals can be a result of a recent outbreak; however, it is unlikely that a large, extensive outbreak would have gone unnoticed. In addition, rainfall in the study area during 2014–2016 had been well below average and the study area, as well as much of South Africa had experienced the worst drought in 23 years [58]. Release of water from the Pongolapoort Dam had not occurred and most of the floodplain pans were empty. Nevertheless, permanent water was still present in the major rivers and some pans, providing breeding habitat for the mosquito vectors. It is therefore likely that the high seroprevalence in all age groups is indicative of a hyperendemic situation [59] with continuous or sporadic viral activity despite the dry conditions, and including recent viral circulation.

Rate or force of infection is a key parameter in modelling of infectious diseases in an attempt to understand the factors determining disease transmission. Our study showed a high seroconversion rate to RVFV in livestock, even during dry conditions, with substantial spatial and temporal variation. Low levels of seroconversion in sentinel sheep and goats have recently been reported in Kenya in areas in which outbreaks are known to occur [33, 34]; however, positive tests were not confirmed using virus neutralization and incidence rates were not reported. In the Kruger National Park, South Africa, seroconversion was demonstrated in 5/227 African buffalo (*Syncerus caffer*), indicating a low annual incidence rate of 1–3% [60]. This was also in the eastern part of the country, adjacent to Mozambique, but outside the tropical zone and in a wildlife area with no livestock and a very low human population. The epidemiology of RVFV in our study area more likely approximates that in other tropical areas of Africa and therefore our results provide useful insight into the dynamics of RVFV transmission in endemic areas, both during interepidemic periods in areas where outbreaks are known to occur, and in areas where outbreaks are not reported. Specifically, the range of seroprevalence and seroconversion rates observed in our study will inform the design of further epidemiological studies in known or suspected endemic areas, as well as the modelling of RVFV transmission in such ecosystems. Knowledge of likely force of infection in endemic areas, and its seasonal variation, will also inform studies to assess the risk of spread of RVFV from such areas via animal movements.

As expected, the highest infection rate was seen during mid to late summer, when water levels are highest and mosquitoes are most abundant. However, despite the fact that rainfall was much higher during the second year of the study, the seroconversion rate was markedly lower, approximately one third of that observed during the first year. It is possible that the drought over the 1-2-year period prior to the study resulted in lower than normal infection rates with a resultant increased number of susceptible animals. This would have increased the number of viraemic animals available to infect mosquitoes, resulting in a higher than usual infection rate during the first year, followed by fewer viraemic animals during the second year. However, the suitable breeding habitat for vectors depends not only on local rainfall, but also on water levels in rivers that are determined by rainfall in their catchment areas. The important mosquito vector species in the study area, and their host preferences, are currently poorly known, and many other factors likely determine their population dynamics, most of which are poorly understood. This study showed very little difference in seroprevalence and seroconversion rate between cattle and goats, suggesting that RVFV is transmitted by one or more mosquito species without strict host preferences.

The differences in seroprevalence and in seroconversion rates between locations could not be readily explained based on geographic location or environmental factors. Two diptanks with high seroprevalence and seroconversion rates (Mbangwini and Hlanjwana, respectively) were close together and near the lower (northern) Pongolo River; however, they could not be distinguished from the other locations close to the river based on environmental or climatic variables and separate rainfall figures were not available for each location; animal management practices were also similar for all locations. Therefore, the risk and rate of RVFV infection are likely determined by a complex interplay of host, vector and environmental factors which our results show may vary substantially within a region, even over small distances. This is consistent with the marked spatial variation in seroprevalence observed in other studies [34, 52, 54, 56]. The lack of an association between seroprevalence (reflecting past seroconversion rate) and subsequent seroconversion rate in our study, as well as the differences in seroconversion rates between the first and second years of the study, also indicates that these factors vary substantially over time. More detailed, fine-scale eco-epidemiological studies to describe the dynamics of these virus-vector-host-environment interactions are required in order to better understand the behaviour of and risk posed by RVFV in such endemic ecosystems.

The seroprevalence in livestock found in this study was much higher than the 12% observed in 1955, the only previous study done in this region [22]. Since then the human and livestock populations in the area have increased tremendously, resulting in a substantial increase in RVFV host density, and it is possible that this has changed the dynamics of viral circulation. Nevertheless, the absence of sheep, the most susceptible livestock species, and the extensive farming system with year-round breeding may be responsible for the fact that large outbreaks do not occur in the area. However, sporadic cases of abortion and neonatal mortality are unlikely to be reported and investigated and may be ascribed to other causes; in addition, disease surveillance in the area is sub-optimal and access to veterinary services is difficult. Therefore, the contribution of RVF to livestock morbidity in the area is unknown and requires further investigation.

Likewise, the potential impact of RVF on human health in the area is unknown. It is generally considered that human infection by RVFV in southern Africa occurs mainly via contact with infected animal tissues from clinical cases and that infection from mosquito bites is less common, as most human cases have a history of exposure to infected animal tissues and the known mosquito vectors tend to be zoophilic [2]. Therefore, the lack of reported cases in livestock in our study area suggest that human exposure to the virus may be low. However, the last published serological surveys done in this region were in the 1950’s, in which 16% of human sera (19/118) collected from locations within in our present study area were protective against RVFV [23]. To our knowledge there has never been a report of clinical RVF in humans in the area; however, the fact that malaria is common there may result in other causes of febrile illness being overlooked. In support of this hypothesis, a recent study in Maputo, 100 km north of our study area, found a 10% RVFV IgG seroprevalence in febrile patients at a primary health care facility, with seroconversion to RVFV in 5%, concluding that undiagnosed RVFV infections occur in that region and that most RVF cases are misdiagnosed as malaria [61]. Therefore, the current status and potential impact of RVFV infection in humans in our study area, and in similar areas along the coastal plain of southern Africa, urgently require further investigation.

### Conclusion

The results of this study show the presence of RVFV antibodies and active seroconversion in domestic ruminants in far northern KwaZulu-Natal, indicating that RVFV is circulating in the area at a rate that varies by location, season and year. Further investigation should be done into the important mosquito vectors in the area, the factors affecting viral circulation and survival, and the impact of the presence of RVFV on animal and human health.

## Supporting information

S1 Data Dataset for cross-sectional and longitudinal studies. (XLSX)

S1 Table Final multiple logistic regression model of factors associated with seropositivity to Rift Valley fever virus in cattle at diptanks in far northern KwaZulu-Natal, June 2016. (DOCX)

S2 Table Multiple logistic regression model of factors associated with seropositivity to Rift Valley fever virus in goats in far northern KwaZulu-Natal, February-April 2017. (DOCX)
