# Supplementary material for: High seroconversion rate to Rift Valley fever virus in cattle and goats in far northern KwaZulu-Natal, South Africa, in the absence of reported outbreaks
Source: PLoS Negl Trop Dis. 2019 May 3;13(5):e0007296. doi: 10.1371/journal.pntd.0007296 (PMC6519843; doi:10.1371/journal.pntd.0007296)
Supplement: S1 Table — (DOCX) [file pntd.0007296.s002.docx]

**Table S1.** Final multiple logistic regression model of factors associated with seropositivity* to Rift Valley fever virus in cattle at diptanks in far northern KwaZulu-Natal, June 2016.

| **Variable and level** | ***OR*** | **95% CI** | ***P*-value** |
| --- | --- | --- | --- |
| **Diptank** |  |  |  |
| Madlakude | 1^†^ |  |  |
| Hlanjwana | 2.15 | 0.84 - 5.5 | 0.110 |
| Masondo | 1.18 | 0.31 - 4.45 | 0.803 |
| Mbangwini | 4.48 | 1.78 - 11.27 | 0.001 |
| Mpala | 1.69 | 0.64 - 4.48 | 0.288 |
| Namanini | 2.20 | 0.8 - 6.06 | 0.129 |
| Ndumo | 2.09 | 0.8 - 5.44 | 0.131 |
| Phelandaba | 4.62 | 1.81 - 11.82 | 0.001 |
| Shemula | 5.83 | 2.3 - 14.76 | <0.001 |
| **Age (years)** |  |  |  |
| <2 | 1.59 | 0.74 - 3.4 | 0.231 |
| 2 – <4 | 1^†^ |  |  |
| 4 – 6 | 2.36 | 1.27 - 4.38 | 0.006 |
| >6 | 1.76 | 0.91 - 3.43 | 0.095 |
| **Sex** |  |  |  |
| female | 1^†^ |  |  |
| male | 0.60 | 0.35 - 1.03 | 0.064 |

* Based on IgG ELISA, confirmed by serum neutralization test in 241/423 samples.

^†^ Reference category

*OR* = odds ratio, CI = confidence interval
