# Supplementary material for: High seroconversion rate to Rift Valley fever virus in cattle and goats in far northern KwaZulu-Natal, South Africa, in the absence of reported outbreaks
Source: PLoS Negl Trop Dis. 2019 May 3;13(5):e0007296. doi: 10.1371/journal.pntd.0007296 (PMC6519843; doi:10.1371/journal.pntd.0007296)
Supplement: S2 Table — (DOCX) [file pntd.0007296.s003.docx]

**Table S2.** Multiple logistic regression model of factors associated with seropositivity* to Rift Valley fever virus in goats in far northern KwaZulu-Natal, February-April 2017.

| **Variable and level** | ***OR*** | **95% CI** | ***P*-value** |
| --- | --- | --- | --- |
| **Age (years)** |  |  |  |
| 0.5 – 1.5 | 1^†^ |  |  |
| >1.5 – 3.5 | 0.96 | 0.29 - 3.26 | 0.954 |
| >3.5 | 1.43 | 0.45 - 4.55 | 0.549 |
| **Sex** |  |  |  |
| female | 1^†^ |  |  |
| male | 0.43 | 0.14 - 1.31 | 0.139 |

* Based on serum neutralization test.

^†^ Reference category

*OR* = odds ratio, CI = confidence interval
